# Supplementary material for: Complexome profiling of the Chlamydomonas psb28 mutant reveals TEF5 as an early PSII assembly factor
Source: Plant Cell. 2025 Mar 17;37(6):koaf055. doi: 10.1093/plcell/koaf055 (PMC12164590; doi:10.1093/plcell/koaf055)
Supplement: koaf055_Supplementary_Data [file koaf055_supplementary_data.zip › psb28_interactive.zip!/plots/intensity/qualityPlots/peptides/PRPL6_Cre09.g415950.t1.2.html]

### Cre09.g415950.t1.2

Trivial name: PRPL6  
SeqCoverage [median]: 19.30  
Mass: 22.23 kDa  
  
 

| Sequence | MissedCleavage | Mass [Da] | wtIntensity | muIntensity | Score |
| --- | --- | --- | --- | --- | --- |
| DLTLNVGYSKPR | 1 | 1361.73 | 948.60 | 1294.45 | 63.41 |
| VAMSQHGLNR | 0 | 1111.56 | 0.00 | 248.90 | 43.28 |
| VLAIPEGLK | 0 | 938.58 | 1561.66 | 0.00 | 65.04 |
|  |  |  | 2510.3 | 1543.4 |  |
